# Effect of dexamethasone administration on gonadal-fertility functions in female albino rats

**DOI:** 10.11604/pamj.2023.44.160.36748

**Published:** 2023-04-05

**Authors:** Fatima Yousif Mohammed, AbdElkarim Abobaker Abdrabo, Samia Mahdi Ahmed, Ahmed Abdulbadie, Zakaria Eltahir, Amar Mohamed Ismail

**Affiliations:** 1 Department of Clinical Chemistry, Faculty of Medical Laboratory Science, Al-Neelain University, Khartoum, Sudan,; 2 Department of Medical Laboratory Technology, Faculty of Applied Medical Sciences, Taibah University, AL-Madinah, Saudi Arabia,; 3 Department of Biochemistry and Molecular Biology, Faculty of Science and Technology, Al-Neelain University, Khartoum, Sudan

**Keywords:** Dexamethasone, lightener, gonadotropins, prolactin, cosmetics, infertility

## Abstract

**Introduction:**

dexamethasone is misused for skin whitening with broad effects on steroidogenesis and ovarian functions. Here in we investigated the impact of dexamethasone administration on gonadotropin Luteinizing Hormone (LH) and Follicle Stimulating Hormone (FSH), prolactin, and ovarian tissues in albino rats.

**Methods:**

in the experimental study, 36 female albino rats weighting (140-162 g) were divided into three groups: control, normal dose received dexamethasone (8.3 μg/kg/day) and high dose (24.9 μg/kg/day), for 30 and 60 days. follicle stimulating hormone, luteinizing hormone, and prolactin (PRL) were measured. Histological ovarian sections were examined.

**Results:**

luteinizing hormone, follicle stimulating hormone and prolactin significantly increased (p-value < 0.05) following dexamethasone treatment compared to control. The ovary sections showed degenerative tissue with necrosis of the Graafian follicles, stromal fibrosis, and vacuolation of the interstitial cells.

**Conclusion:**

the study concludes that dexamethasone administration has a potentially adverse effect on gonadotropin, prolactin, and ovarian follicle cells in female albino rats.

## Introduction

Skin whitening is the misuse of skin lightening agents such as dexamethasone and glutathione, it is a cosmetic process associated with social and public health problems in the last two decades [1]. Abuse of skin lightening products is widespread in Africa and Asia, ranging from 25% to 96% [2]. Most used skin whitening types are hydroquinone, topical steroids, mercurials, kojic acid, and sometimes products like battery fluid and cement, which are administrated as pills, injections, creams, soaps, and natural homemade skin-bleaching products are potentially harmful [1,3]. Hence, skin lighting agents are predisposing factors to skin cancer, their cosmetics marketing are increased [4]. Dexamethasone is a medication used as immunosuppressive, anti-inflammatory, and for treating many skin diseases [5,6]. Dexamethasone locally (Alnagma) is misused as a cosmetic for gaining weight and whitening skin, regardless of perception, doses, storage, and adverse effects [7]. Abuse of dexamethasone increased luminal steroid hormones with hyperplasia of theca and granulosa cells [7-9]. To our knowledge, no study has investigated an adverse effect of dexamethasone on the gonadal-fertility functions. Therefore, this study was carried out to investigate the impact of dexamethasone misuse of gonadotropins, prolactin, and ovarian sections in albino rats.

## Methods

**Preparation of drug:** dexamethasone tablets (0.5 mg) were purchased from a local pharmacy in Omdurman. The cosmetics doses were prepared after crashed into powder, then weighing, and dissolved in 3ml of distilled water as a stock solution, later was diluted to the low and high doses.

**Study design:** in experimental randomized study, a total of 36 female albino rats with body weight range (140-162 g) and age range (3-4 months) were used. The rats were isolated before one month of the experiment for adaptation, then divided equally into three groups: group I control group which was given a placebo (distilled water) orally, group II and III treated with dexamethasone orally with a low dose (8.3 μg/kg/day) and high dose (24.9 μg/kg/day) respectively, after 30 days (experiment 1) and 60 days (experiment 2) rats were sacrificed. The specimens of the ovary were immediately fixed in 10% neutral buffered formalin and processed for histopathology. Blood samples were collected. Serum was obtained after centrifugation at 3000 rpm. Fertility hormones (LH, FSH, and Prolactin) were measured.

**Ethical considerations:** the animal experiment was designed and conducted under the declaration of Helsinki. Ethical approval was obtained from the King Abdul-Aziz University, Pharmacy College (Reference No.546-20).

**Estimation of fertility hormones:** in brief, according to the manufacturer FSH (catalog no: MBS2502190) (my biosource, Inc. Germany) was measured using the double-sandwich ELISA technique. A micro-ELISA plate was pre-coated with an antibody specific to rat FSH, and samples were added to each labeled well. Biotinylated detection antibody specific for rat FSH and avidin-horseradish peroxidase (HRP) conjugate were added. Free components were washed. The substrate solution was added. The blue color formed is proportional to FSH level. The enzyme-substrate reaction was terminated by stop rection solution. Luteinizing hormone (catalog number: MBS729873) and prolactin (catalog number: MBS727546) (My biosource, Inc. Germany) were measured using a competitive enzyme immunoassay technique utilizing a polyclonal anti-LH antibody and an LH-HRP conjugate, anti-PRL antibody and a PRL-HRP conjugate. Sample and buffer were incubated together with LH, PRL-HRP conjugate in a pre-coated plate, after incubation plate was washed. Then substrate was added. The blue-colored formed is inversely correlated with LH and prolactin concentration. Finally, the intensity of color was assessed by fully automated ELISA dynex best 2000 analyzer, serial number (835485).

**Histological methods:** after anesthesia with diethyl ether, the rats were dissected. Specimens of the ovary were collected and immediately fixed in 10% neutral buffered formalin. The ovary was embedded in paraffin wax, sectioned at 5µm, diameter, and stained routinely with hematoxylin and eosin (H and E) [10].

**Statistical analysis:** it was carried out using Statistical Package for (SPSS) software version 21.0 (SPSS ver. 21.0, Inc., Chicago, IL, USA). Data were expressed as mean ± standard error of the mean (SEM). Independent t-tests and Analysis of variance (ANOVA) were employed to compare the mean between groups. A significant difference was considered as a p-value≤0.05.

## Results

**Figure 1:**
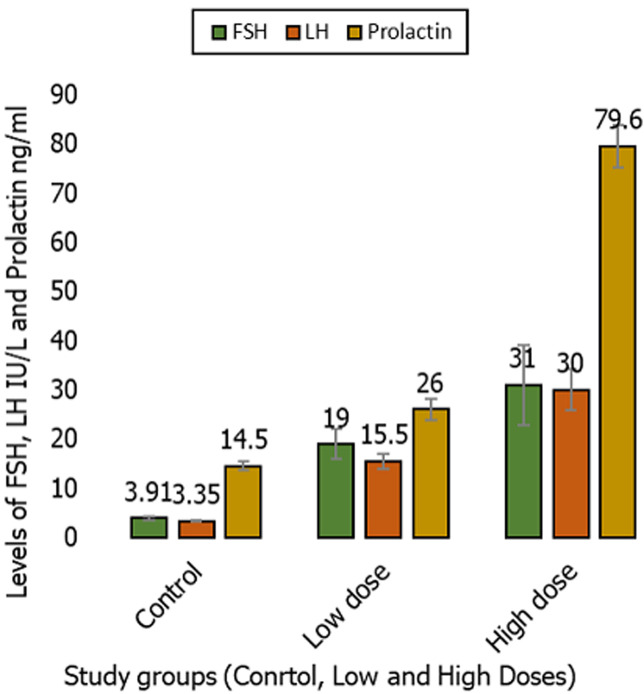
comparison of the mean follicle stimulating hormone, luteinizing hormone and prolactin levels of study groups, following treatment with dexamethasone for 30 days

**Figure 2:**
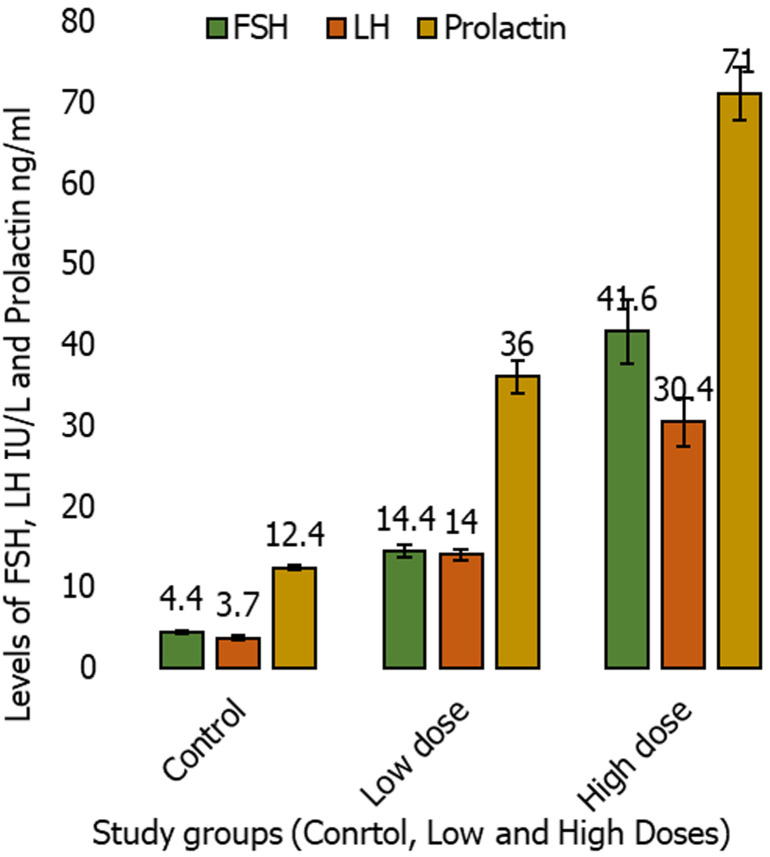
comparison of the mean follicle stimulating hormone, luteinizing hormone and prolactin levels of study groups, following treatment with dexamethasone for 60 days

**Fertility hormones results:** administration of dexamethasone for 30 days, there were significant increases in the mean concentration of FSH in the group treated with low doses (19.0 ± 3.10 IU/L), and high doses group (31.4 ± 8.37 IU/L) when compared to the control group (3.91 ± 0.33 IU/L) with (p < 0.05). Meanwhile, there were significant increases in the mean concentration of LH in the group treated with low dose (15.3 ± 1.60 IU/L), and high dose (30.0 ± 4.18 IU/L) in comparison with the control group (3.35 ± 0.21 IU/L) with (p < 0.05). Moreover, there were significant increases in the mean concentration of PRL of group administrated low (26.0 ± 2.21 ng/mI), and high dose (79.6 ± 4.30 ng/mI) when compared to control group (14.5 ± 0.95 ng/ml) with (p < 0.05), presented in Figure 1. Following administration of dexamethasone for 60 days, there were significant increases in the mean concentration of FSH in the group treated with low dose (14.4 ± 0.75 IU/L), and high dose (41.6 ± 3.91 IU/L) than the control group (4.11 ± 0.28 IU/L) with (p < 0.05), and there were significant increases in the mean concentration of LH in the group that received a low dose (14.0± 0.72 IU/L), and a high dose (30.4 ± 2.95 IU/L) in comparison to the control group (3.70 ± 0.22 IU\L) with (p < 0.05). Moreover, there were significant increases in the mean concentration of PRL in low dose treated group (36.0 ± 2.02 ng/mI), and in high dose (71.0± 3.31 ng/mI) when compared to control group (12.4 ± 0.34 ng/mI) with (p < 0.05) presented in Figure 2.

**Figure 3:**
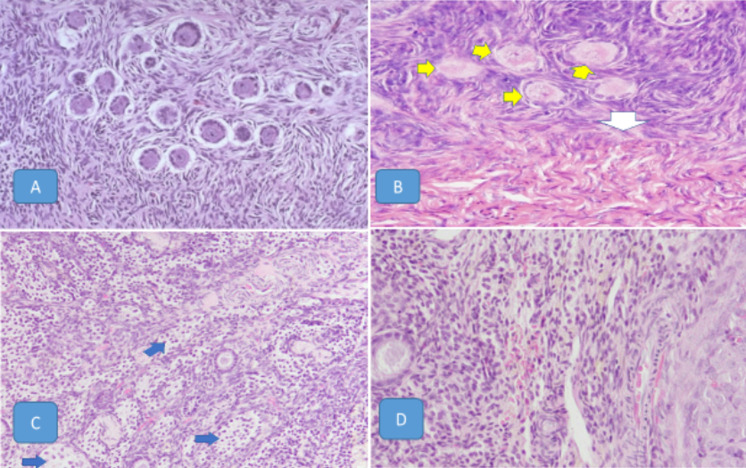
histological section of ovary sections stained with (H and E): A) control group; B) treated with the low dose dexamethasone for 60 days; C) treated with a high dose for 30 days; D) treated with high dose for 60 days

**Histological results:** histological section of ovary stained with (H and Ex40, 20,10, 40); A) control group showing normal ovarian follicle numbers with normal blood vessels and normal interstitial cells; B) treated with a low doses (8.3µg/kg/day) for 60 days, shows degenerative ovary with necrotic Graafian follicles (yellow arrows), and stromal abnormal reactivity (white arrow); C)treated with a high dose (24.9 μg/kg/day) for 30 days, shows vacuolation of the interstitial cells covering the background replacing the stromal tissue (blue arrows), ovary showing marked reduction in ovarian follicle numbers with congestion of blood vessels and marked degeneration of interstitial cells and some follicles; D) treated with high dose dexamethasone (24.9 μg/kg/day) for 60 days, ovary showing marked reduction in ovarian follicle numbers with congestion of blood vessels, marked degeneration of interstitial cells, and some follicles with hemorrhage (Figure 3).

## Discussion

The data of the present study showed that administration of dexamethasone in albino rats significantly increased LH, FSH, and prolactin. Meanwhile, leads to a marked reduction in follicle numbers of ovaries, degeneration of interstitial cells, and some follicles with hemorrhage, which is following previous studies that treatment with dexamethasone increases gonadotropin and induces apoptosis. The present study revealed a significant increase in gonadotropin following administration of dexamethasone for 60 days compared to the control group. Other studies have suggested that dexamethasone increases the level of LH and FSH [9,11]. On the other hand, rats treated with dexamethasone significantly decrease patterns of ovarian estrogen and progestin secretion [12], consequently leading to higher gonadotropins, which is probably due to negative feedback inhibition from gonads [13,14]. Meanwhile, dexamethasone disrupts cholesterol transporting protein in the gonads [15]. Resulting in low estrogens which increases gonadotropin. Despite some studies that have reported that low-dose dexamethasone treats hyperandrogenic infertility and unresponsive ovary to gonadotropin invitro fertilization (INF), therefore these contradictions may be due to the exact dose and the duration of treatment. Concurrent with a previous study that prolactin levels were elevated in the dexamethasone-treated group [16], however the mechanism of hyperprolactinemia following administration of dexamethasone is unknown and higher prolactin can cause infertility and bone loss [17]. The present study demonstrated that dexamethasone causes degenerative ovary with necrotic Graafian follicles, vacuolation of the interstitial cells, marked reduction in ovarian follicle numbers with congestion of blood vessels, degeneration of interstitial cells, and follicles hemorrhage. Therefore, indicates inhibition of reproduction [18] and ovarian follicles damage following dexamethasone abuse for skin lighting, consequently infertility in female albino rats.

**Limitations:** in the present study, we did not measure the female-gonadal estrogens (estradiol and progesterone), which might help in the explanation of the mechanisms behind increasing level of the gonadotropins (LH and FSH). Therefore, similar study set up is recommended to investigate the effect of dexamethasone misuse on ovarian-estrogens.

## Conclusion

The data of present suggests that dexamethasone administration in female albino rats increases gonadotropins and prolactin levels. Moreover, induces ovarian follicle damage, these results indicate possible evidence that administration of dexamethasone for skin lighting causes infertility in females. Therefore, the present study encourages further investigation to elucidate insight into the mechanisms associated with infertility.

### What is known about this topic

- *The dexamethasone administration as cosmetics is harmful to female-gonadal functions and induces ovarian follicles damage*.

### What this study adds

- *Oral dexamethasone misused as skin lightener significantly increased gonadotropins, prolactin and induces ovarian cells damage;*
- *A severity of the potential adverse effects depends on the doses and the duration of dexamethasone misused*.
